# Coronary functional assessment in non-obstructive coronary artery disease: Present situation and future direction

**DOI:** 10.3389/fcvm.2022.934279

**Published:** 2022-08-23

**Authors:** Changlin Zhai, Hongyan Fan, Yujuan Zhu, Yunqing Chen, Liang Shen

**Affiliations:** ^1^Department of Cardiology, Affiliated Hospital of Jiaxing University, Jiaxing, China; ^2^Department of Infectious Diseases, Affiliated Hospital of Jiaxing University, Jiaxing, China

**Keywords:** coronary functional assessment, INOCA, MINOCA, microvascular dysfunction, vasomotion abnormality

## Abstract

Non-obstructive coronary artery disease (CAD), which is defined as coronary stenosis <50%, has been increasingly recognized as an emerging entity in clinical practice. Vasomotion abnormality and coronary microvascular dysfunction are two major mechanisms contributing to the occur of angina with non-obstructive CAD. Although routine coronary functional assessment is limited due to several disadvantages, functional evaluation can help to understand the pathophysiological mechanism and/or to exclude specific etiologies. In this review, we summarized the potential mechanisms involved in ischemia with non-obstructive coronary arteries (INOCA) and myocardial infarction with non-obstructive coronary arteries (MINOCA), the two major form of non-obstructive CAD. Additionally, we reviewed currently available functional assessment indices and their use in non-obstructive CAD. Furthermore, we speculated that novel technique combined anatomic and physiologic parameters might provide more individualized therapeutic choice for patients with non-obstructive CAD.

## Introduction

Angina with non-obstructive coronary artery (defined as lesions with <50% stenosis) has been recognized as a frequent problem encountered in clinical practice (1, 2). It is reported that probably 60–70% of patients who underwent coronary angiography due to angina pectoris and evidence of myocardial ischemia do not have obstructive coronary disease (3–7), a condition recently termed ischemia with non-obstructive coronary arteries (INOCA). Myocardial infarction (MI), the most serious form of coronary artery disease (CAD), might also occurred in coronary artery without obvious occlusion, which now named as myocardial infarction with non-obstructive coronary arteries (MINOCA) (8). Previous studies demonstrated that MINOCA can be found in 5–10% of all patients with MI (9–13).

Although increasing attention have been paid to non-obstructive CAD, the mechanisms are largely unknown, and the diagnosis might be underestimated in the real world. Therefore, it is crucial to identify non-obstructive CAD to provide appropriate management strategies. Coronary functional assessment has emerged as the “golden standard” to optimize the treatment of CAD (14). Besides, coronary functional assessment can identify the underlying pathophysiological mechanisms in non-obstructive CAD. In this review, we discussed potential mechanisms involved in two widely accepted form of non-obstructive CAD, MINOCA and INOCA, as well as coronary physiologic assessment techniques and their application in non-obstructive CAD.

## Definition of MINOCA and INOCA

MINOCA is a syndrome characterized by clinical signs of myocardial infarction with no remarkable stenosis (≥50%) of coronary artery on angiography. According to the latest scientific statement published by the AHA, the diagnosis of MINOCA should meet the following criteria: (1) AMI meeting the “Fourth Universal Definition of Myocardial Infarction” criteria; (2) no artery stenosis ≥50% on coronary angiography in any major epicardial vessel; (3) no specific alternate diagnosis for the acute clinical presentation (8).

INOCA is a condition defined as cardiac ischemia in the absence of obvious obstruction of epicardial coronary artery diameter reduction (≥50%) by coronary angiography. Accordingly, ACC published a uniform definition of INOCA as follows: (1) persistent (several weeks or longer) symptoms suggesting ischemic heart disease; (2) objective evidence of myocardial ischemia from ECG or other cardiac imaging test, such as echocardiography, magnetic resonance imaging, nuclear imaging, or spectroscopy; (3) absence of flow-limiting obstruction by coronary angiography as defined by epicardial coronary artery stenosis ≥50% or fractional flow reserve <0.8 (5). It is worthy to note that the diagnosis of INOCA can be established when other myocardial ischemia mechanisms such as cardiomyopathy, aortic stenosis, cardiac infiltrative diseases, systemic inflammatory and autoimmune disease, primary metabolic abnormalities and myocardial bridging are excluded (4).

## Pathophysiology of MINOCA and INOCA

The potential underlying pathophysiological mechanisms for MINOCA include: (1) atherosclerotic causes of myocardial necrosis, including plaque rupture, plaque erosion, and calcific nodules, collectively referred to as plaque disruption; (2) non-atherosclerotic causes, such as epicardial coronary vasospasm, coronary microvascular dysfunction, coronary embolism/thrombosis, and spontaneous coronary artery dissection (8, 15).

Generally, plaque disruption cannot be accurately determined or distinguished by coronary angiography, it can only be definitively diagnosed with optical coherence tomography (OCT) or, to a lesser extent, with intravascular ultrasound (IVUS) (16–18). Nowadays, OCT has been an essential diagnostic modality for MINOCA (19). OCT can identify the hallmark of a culprit lesion, including plaque disruption and thrombus. Plaque rupture is characterized by the presence of fibrous cap discontinuity with a cavity formation within the plaque, which often caused by inflammation and occurred in thin fibrous cap (<65 μm) (20, 21). Plaque erosion is characterized by the presence of attached thrombus overlying an intact and visualized plaque, and the thrombus is always regarded as “white thrombus” (22). Calcified nodule is defined as signal or multiple regions of calcium that protrudes into the arterial lumen, with fibrous cap disruption forming sharp, protruding edges, which is less common than plaque rupture or erosion in patients with acute coronary syndrome (16, 23). As mentioned above, plaque disruption can lead to thrombus formation, which might cause distal embolization and coronary spasm, moreover, inflammation might directly contribute to the necrosis of cardiomyocyte (24) and stimulate coronary spasm (25) or microvascular dysfunction (26) as well, eventually contribute to the development of MINOCA.

Coronary artery spasm refers to intense constriction (>90%) of an epicardial coronary artery and/or microvascular, leading to limited myocardial blood flow (27). Coronary spasm is firstly reported in patients with obstructive CAD (28), however, it is widely occurred in patient with non-obstructive coronaries (29). It is primarily presented as unstable angina with dynamic ST-segment elevation pattern on ECG, while prolonged spasm can also result in myocardial infarction.

Microvascular dysfunction is defined as coronary microvascular (vessels <0.5 mm diameter) structural remodeling or functional dysregulation with reduced myocardial perfusion, which is characterize by impaired coronary flow reserve and abnormal coronary microvascular resistance indices (30). Structural microvascular dysfunction is associated with a decrease of microvascular conductance and impaired oxygen delivery capacity. The decreased microvascular conductance is due to the remodeling of small-sized coronary arterioles and the impaired oxygen delivery is the result of loss of myocardial capillary density (31). In contrast, functional microvascular dysfunction usually represents an impaired flow-mediated vasodilation, which can be divided into endothelium-dependent or-independent. Coronary microvascular dysfunction (CMD) is prevalent in a broad spectrum of cardiovascular diseases, and it can be categorized into CMD without atherosclerosis, CMD with non-obstructive atherosclerosis and CMD with obstructive atherosclerosis. In patients with non-obstructive atherosclerosis, CMD might occur more frequently in patients with cardiometabolic disease (i.e., metabolic syndrome, obesity, and diabetes mellitus), chronic kidney disease, and heart failure with preserved ejection fraction (HFpEF), and is disproportionately female (32, 33). Previous studies showed microvascular dysfunction in patients with MINOCA, however, whether it is the reason of myocardial infarction or just as the consequence of MI has not been fully elucidated. Further researches are needed to further investigate the role of microvascular dysfunction in MINOCA.

Coronary embolism/thrombosis is defined as coronary artery obstruction caused by embolus and/or thrombus, which interrupt the oxygen supply to myocardial and cause myocardial infarction (34, 35). In some cases, spontaneous thrombolysis of complete thrombosis might occur and leading to the occlusion of microvascular. Coronary embolism/thrombosis can also be the result of hypercoagulable disorders, such as thrombotic thrombocytopenic purpura (TTP) (36) and antiphospholipid syndrome (37).

Spontaneous coronary artery dissection is a non-atherosclerotic, non-traumatic cause of acute coronary event with the development of hematoma within the tunica media (38). It has emerged as an important cause of MI, particularly among younger women (39). So far, the precise mechanism of SCAD is not entirely known and the exact incidence of SCAD might be underestimated. Similar to plaque disruption, the diagnosis of SCAD mostly depends on intravascular imaging such as IVUS and OCT (40–42).

Recently, myocardial bridging, a congenital coronary anomaly, has also been investigated and is emerging as an important player in determining MINOCA (43). In patients with MINOCA, myocardial bridging is an independent predictor of positive acetylcholine (Ach) test and the coexistence of myocardial bridging and a positive Ach test in frequent, indicating a role for coronary spasm superimposed to myocardial bridging in MINOCA patients (44).

Despite many causes of MINOCA have been investigated, the exact mechanisms of MINOCA is largely unknown. We speculate that inflammation plays a vital role during MINOCA. As we known, inflammation is a major trigger of plaque rupture (45), inflammation reaction might not limit to the ruptured plaque, myocardium adjacent to the plaque or even the whole heart may be in an inflammatory state, which results in the death of cardiomyocytes. Moreover, spontaneous thrombolysis, an established cause of MINOCA, might lead to ischemia/reperfusion injury, which further promotes the generation of reactive oxygen species (ROS), inflammation and finally cell death (ie, necrosis, apoptosis or ferroptosis) (46, 47), which might explain the occurrence of myocardial infarction with no obvious obstruction of coronary artery.

INOCA shares some similar pathophysiologic mechanisms with MINOCA. The most common mechanisms of INOCA are microvascular dysfunction and epicardial coronary artery spasm (4). The common risk factors of INOCA including female, smoking, obese, hypertension and diabetes. Unlike MINOCA, no microcirculatory occlusion or persistent coronary spasm might be observed in the context of INOCA.

## Coronary functional assessment methods

Numerous techniques for the assessment of coronary physiologic function have been developed. Fractional flow reserve (FFR) is most widely accepted index for the physiological function evaluation of coronary disease, which is defined as the ratio between mean distal pressure and mean aortic pressure at the stage of maximal hyperemia typically stimulated by adenosine. Regadenoson, a selective A2a receptor agonist, has also been demonstrated as a viable alternative to intravenous adenosine for measuring FFR (48–50). FFR ≤ 0.80 is recommended as the cut-off value for the diagnosis of functionally relevant coronary disease (51). Although FFR is recommended as a class Ia indication in myocardial revascularization among chronic coronary syndromes (CCS) patients (14), the utilize of FFR is relatively limited in the real world mainly due to prolonged procedural time, expensive cost and the need of hyperemic agents. Hence, a number of alternative methods have been developed and used in clinical practice. Among which, submaximal hyperemic physiological indices (e.g., contrast-based FFR) and resting indices, such as instantaneous wave-free ratio (iFR), resting full-cycle ratio (RFR), diastolic pressure ratio (dPR) have been utilized in clinical trials or practice due to their advantage of no hyperemic drugs needed (52, 53). iFR is the ratio of mean distal pressure and mean aortic pressure during the period of diastolic wave-free, recent studies have demonstrated that iFR is non-inferior to FFR in terms of clinical outcomes in patients with CAD, the recommended cut-off value of iFR is 0.89 (54, 55). RFR and dPR are another two adenosine-free functional assessment methods, which is calculated as the minimal distal pressure with reference to the aortic pressure during five entire cardiac cycles, and as an averaged Pd/Pa ratio during a part or the entire diastolic period without selection of a wave-free period respectively. Both methods have been investigated and revealed a significant correlation with iFR (56, 57), indicating their promising value in the treatment of CAD patients. Contrast-based FFR (cFFR) is another valuable adenosine-free index, which is measured as the mean distal coronary pressure divided by aortic or proximal coronary pressure during submaximal hyperemia with intracoronary injection of contrast. Previous studies had shown that cFFR provided better diagnostic performance than Pd/Pa or iFR for predicting FFR (58, 59).

Nevertheless, prolonged procedural time, extra cost and inevitable wire-related complications remain big challenges to the routine use of these resting functional assessments. Therefore, quantitative flow ratio (QFR) was discovered and almost perfect successfully settled these limitations. QFR is a novel, wireless, vasodilator-free coronary functional assessment method based on computational modeling of 3-dimensional (3D) quantitative coronary angiography (QCA) and Thrombolysis in Myocardial Infarction (TIMI) frame counts (60). A plenty of clinical trials have illustrated the clinical feasible and diagnostic accuracy comparable to FFR (60–63). Most recently, the results of FAVOR III China, a multicenter, blinded, randomized, sham-controlled trial, showed that compared with angiography guidance, a QFR-guided strategy improve 1-year clinical outcomes among patients undergoing PCI (64). It is believed that QFR would be a promising tool to guide the clinical practice in patients with CAD.

Similar to QFR, coronary angiography-derived FFR (FFR_angio_), another method to calculate FFR just depends on angiogram with no coronary pressure wire of hyperemic agent, has also been shown to have a high diagnostic performance compared with conventional FFR (65, 66).

As discussed above, functional assessment shed light on culprit lesions, while intracoronary imaging, including OCT and IVUS, optimize the intervention strategy. Previous studies have shown that both OCT- and IVUS-guided PCI can reduce target vessel failure compared with angio-guided PCI (67, 68). Hence, it would be plausible to combine functional assessment technology with intracoronary imaging to maximize the benefit of PCI. Excitingly, OCT-derived FFR (OFR, an OCT-based method for the functional assessment of coronary stenosis based on computational fluid dynamics) has been developed and investigated in clinical trials, and the results showed that OFR can be a feasible and alternative method for physiology assessment as well as coronary morphology (69–71). Ding et al. (72) demonstrated that post-PCI OFR showed good diagnostic concordance with post-PCI FFR. Recently, IVUS-derived FFR (UFR) was developed as well, and also was found to have a good agreement and strong correlation with FFR, suggesting it is a valuable method to identify myocardial ischemia (73, 74). Moreover, coronary CT-derived FFR (FFR_CT_), a non-invasive evaluation of CAD that provides a combined anatomic and physiologic assessment, has emerged as an alternative method to FFR for decision-making and identification of targets for revascularization in patients with extensive CAD (14).

Compared with epicardial coronary arteries, coronary microvascular might play a more crucial role in the physiology of heart. Nowadays, several methods have been established for the assessment of microvascular function (75). Coronary flow reserve (CFR) is one of the methods to evaluate the function of microcirculation. CFR is the ratio of vasoactive regents (e.g., adenosine) stimulated-hyperemic blood flow divided by resting blood flow. It can be calculated using thermodilution or Doppler flow velocity (76). The generally acknowledged cut-off value of CFR is 2.0 or 2.5 depending on methodology, numerous studies have shown that there is a close association between low CFR and poor prognosis (77–79).

CFR assessment can be divided into an endothelium-independent component and endothelium-dependent coronary flow. After non-endothelium-dependent CFR assessment acquired *via* adenosine administration, the endothelium-dependent epicardial diameter and microvascular should be evaluated with the injection of acetylcholine (Ach), also known as provocation test. A positive provocation test should include three criteria: angina symptoms, ischemic ECG changes and severe vasoconstriction (>90%) of the epicardial vessel (80). In some cases, angina might occur in the absence of angiographically evident spasm, even without ST-segment changes, which might support the diagnosis of microvascular angina.

However, CFR cannot specifically reflect the microvascular function when there is a significant epicardial disease, therefore, it is reasonable to use the index of microvascular resistance (IMR) to correctly assess the function of microvascular. IMR is calculated as the product of distal coronary pressure at maximal hyperemia multiplied by the hyperemic mean transit time (81). IMR ≥ 25 is regarded as the cut-off value of microvascular dysfunction (82). According to the 2019 ESC guideline for CCS, in patients either angiographically normal or have moderate stenosis with preserved iFR/FFR, CFR and/or IMR are recommended as IIa indication (14). Hyperemic myocardial velocity resistance (HMR), a Doppler-based index, calculated by dividing intracoronary pressure by hyperemic flow velocity, also can reflect the microvascular function. HMR > 1.9 is regarded as the cut-off to predict microvascular angina (83).

Regardless, some disadvantages exist in both methods, such as prolonged procedural time, extra medical cost, stable injection technology needed and hyperemic medicine required, which are similar to traditionally epicardial coronary physiology evaluation devices. As a result, continuous intracoronary thermodilution and angiography-derived index of microcirculatory resistance (angio-IMR) have been discovered to overcome these limitations. Continuous intracoronary thermodilution is a novel technique to quantify absolute coronary flow (Q) and resistance (R) with a continuous infusion of saline to instead adenosine (84). Briefly, Q is calculated as 1.08 × (Ti/T) × Qi, R is calculated as Pd/Q. Ti stands for the temperature of the infused saline, while T is the temperature of the mixture of blood and saline, Qi is pre-specified flow rate (20 ml/min in LAD and LCX, 15 ml/min in RCA), Pd is the distal pressure of the target coronary. It has been demonstrated that continuous thermodilution is feasible and safe in patient with INOCA (85), but the exact diagnostic and prognostic value and the optima cut-off value need to be further determined.

Angio-IMR is another novel microcirculatory functional assessment technique, which does not need pressure wire, hyperemic medicine and thermodilution method (86). It is defined as estimated Pa_hyp_× [L/(κ × V_diastole_)], Pd_hyp_ is the mean pressure at the distal position when maximal hyperemia, L represents the length from the inlet to the distal position, V_diastole_ is the mean flow velocity at the distal position at diastole. Recently, angio-IMR has been investigated in CAD (87) and patients with ST-segment elevation myocardial infraction (STEMI) (88), and the results showed that angio-IMR has a high correlation with wire-derived IMR and high accuracy to predict microvascular dysfunction (88).

Nevertheless, these methods are all depend on coronary angiography, in other words, are invasive. It would be more attractive to use non-invasive methods to estimate the index of microvascular function. So far, several non-invasive CFR assessment techniques have been developed, including transthoracic Doppler echocardiography (TTDE) (89), myocardial contrast echocardiography (MCE) (90), positron emission tomography (PET) (91), and cardiac magnetic resonance (CMR) (92). TTDE, PET and CMR have been recommended as IIb indication for the detection of microcirculatory dysfunction (14). The detailed information as well as advantages and disadvantages of various invasive and non-invasive methods are summarized in Table 1.

**Table 1:** Characteristics of various coronary functional assessment indices.

**Functional indices**	**Cut-off value**	**Pros**	**Cons**
FFR	0.8	Golden standard of PCI	Prolonged procedural time, expensive cost and the need of hyperemic agents
iFR	0.89	Non-inferior to FFR, independent of hyperemic agents	Discordance with FFR in specific patients
RFR	0.92	Non-inferior to iFR, independent of hyperemic agents	Need to continuously analyze 5 cycles
dPR	0.89	High concordance with iFR, independent of hyperemic agents	Narrow value range
QFR	0.8	Wireless, independent of hyperemic agents, short-time	Need high quality angiography imaging
FFR_angio_	0.8	Similar to QFR	Similar to QFR
FFR_CT_	0.8	Non-invasive, low risk, inexpensive	Depend on resolution and imaging quality, needs for heart rate control
OFR	0.8	Simultaneously evaluate anatomic and physiologic changes	More expensive, add the use of contrast and the risk of complication
UFR	0.8	Similar to OFR, but with lower resolution	More expensive, depend on guidewire
CFR	2.0	Provide information of microvascular function	Affected by epicardial coronary and resting hemodynamics
IMR	25 U	Reflect the function of microcirculation, less influenced by hemodynamics	Need hyperemic agents
HMR	1.9 mmHg/cm·s	Similar to IMR	Hyperemic agents dependent
Angio-IMR	40 U	Independ on guidewire and hyperemic agents	High quality image needed
Continuous intracoronary thermodilution	320 ml/min for Q and 487 WU for R	Independent of hyperemic agent and operative skill	Relatively complex in measurement
TTDE	2.0	Non-invasive, low cost and good reproducibility	Require extensive training, more feasible in LAD, less satisfactory in other coronary
PET	2.3 mL/min/g	High sensitive and non-invasive	Less availability and costly
CMR	1.5	Non-invasive, high resolution and without radioactivity	Less reproducibility

PCI, percutaneous coronary intervention; FFR, fractional flow reserve; iFR, instantaneous wave-free ratio; RFR, resting full-cycle ratio, dPR, diastolic pressure ratio; QFR, quantitative flow ratio; FFR_angio_, angiography-derived FFR; FFR_CT_, coronary CT-derived FFR; OFR, optical coherence tomography-derived FFR; UFR, intravascular ultrasound-derived FFR; CFR, coronary flow reserve; IMR, index of microvascular resistance; HMR, hyperaemic myocardial velocity resistance; Angio-IMR, angiography-derived IMR; TTDE, transthoracic Doppler echocardiography; LAD, left anterior descending; PET, positron emission tomography; CMR, cardiac magnetic resonance.

## Coronary functional assessment in non-obstructive CAD

For patients with persistent angina but angiographically normal or moderate stenosis with preserved iFR/FFR, guidewire-based CFR and/or IMR measurements is recommended as IIa indication to detect microvascular angina. Intracoronary provocation test is also a class IIa recommendation to identify coronary spasm in patients with non-obstructive lesions on coronary arteriography. Additionally, intracoronary injection of acetylcholine may also be considered to assess microvascular vasospasm (14).

As the guideline recommended, preserved iFR/FFR is essential in the diagnose of microvascular angina with angiographically moderate coronary stenosis. In other words, we might need to perform iFR/FFR measure to rule out pseudo-non-obstructive CAD with mild to moderate stenosis (93). Since previous studies had revealed that both RFR and dPR were closely correlated with iFR, and cFFR was superior to iFR for predicting FFR, it is reasonable to use these indices to exclude actually obstructive coronary with <50% stenosis, despite there is a lack of large clinical trials to investigate the role of these methods in the context of INOCA. QFR, an angiography-based approach, might be more promising in the detect of flow limited coronary lesions with less cost, time and complications associated with pressure wire and hyperemic drugs. In a *post-hoc* analysis of patients without significant angiographically stenosis, QFR value ≤ 0.80 was showed to be strongly associated with major adverse cardiovascular and cerebrovascular events in long-term follow-up (94). However, precisely calculate QFR depends on the high quality of angiography images, besides, microcirculatory resistance and coronary flow velocity might affect the consistency between QFR and FFR (95). Larger studies are needed to further investigated the role of QFR in non-obstructive CAD. FFR_CT_ is another possible alternative method to FFR, due to its non-invasive characteristic with comparable property to invasive FFR (96, 97). However, it is reported that FFR_CT_ value ≤ 0.80 was found in the distal coronary artery segment in asymptomatic male marathon runners with no coronary stenosis (98), therefore, it is important to interpret the result of FFR_CT_ individually. Most recently, a prospective multicenter observational study, the China CT-FFR Study-2, has conducted to investigate the prognostic value of FFR_CT_ in patient with non-obstructive CAD, which will provide us with a comprehensive risk stratification of FFR_CT_ for non-obstructive CAD (99).

Microvascular angina account for a large proportion of patients with INOCA, CFR and IMR are widely accepted methods to assess microvascular dysfunction (78). However, both CRF and IMR need hyperemic stimulus, which limits their routine use in clinical practice. Continuous intracoronary thermodilution and AngioIMR have been investigated in patients without obvious obstructive coronary stenosis. Rivero et al. (84) and Konst et al. (85) both reported that IMR derived from continuous intracoronary thermodilution is an accurate index to diagnosis of coronary microvascular dysfunction. The value of continuous intracoronary thermodilution for the assessment of microcirculatory dysfunction was also verified in patients with diabetes (100). Most recently, the role of AngioIMR in INOCA was examined as well, and the result revealed that AngioIMR is moderate correlation with HMR and has high accuracy in predicting microcirculatory dysfunction (87). Provocation test with acetylcholine might should also be considered as functional test to identify whether epicardial or microvascular vasospasm exit in patient with suspected INOCA.

Unlike INOCA, no guideline recommended routine coronary functional assessment in acute myocardial infarction. However, recent evidence indicate that functional assessment is crucial to identify the underlying pathophysiological mechanisms and provide individual treatment strategy to MINOCA. In stable chest pain patients, up to 25% ones with 30–50% stenosis have functionally significant stenosis when assessed by FFR (93), which might also apply to MINOCA. However, limited data yet evaluate the role of FFR in MINOCA. According to the AHA statement, FFR may be considered in select patients with borderline obstructive lesion, and only if FFR >0.80 can be diagnosis as MINOCA (8). Similar to INOCA, it is reasonable to use resting physiological indices, despite no supported data, to instead of FFR to avoid the use of hyperemic agents. Recently, Li et al. (101) reported the use of QFR in a patient with MINOCA and proposed QFR might be an efficient approach in the context of MINOCA. Nevertheless, more studies are needed to determine the number of patients with suspected MINOCA who have functionally significant stenosis.

Microcirculatory dysfunction is also regarded as an important cause of MINOCA. To date, no clinical trials investigated the role of conventional wire-dependent CFR and IMR in MINOCA, but most recently, angiography-derived IMR has been investigated in MONICA. The results showed that angiography-derived IMR (caIMR) was a strong predictor of clinical outcome among patients with MONICA, and high caIMR (>43) was an independent predictor of MACE (102). Nonetheless, we still have no idea about the causal relationship between microcirculatory resistance and MINOCA.

CMR is one of the widely used technique to assess myocardial perfusion (103). It allows visualization of transmural myocardial flow and assessment of microvascular function in patients suspected with microvascular angina (104). Meanwhile, CMR is crucial for the diagnosis of MINOCA, due to its perfect ability to exclude acute myocarditis and Takotsubo cardiomyopathy (105). Moreover, CMR is non-invasive and non-radioactive, therefore, CMR is an ideal method to evaluate the microcirculatory dysfunction of MINOCA, especially high-resolution late gadolinium enhancement CMR (106).

Traditionally, provocation test is not routinely used in the context of MI due to multiple safety concerns, such as malign arrhythmias. However, prolonged vasospastic episodes can result in MINOCA and patients with coronary spasm-induced MINOCA might have a higher risk of cardiac death, recurrence of ACS and worse angina (29). Actually, provocation test with Ach has been demonstrated as a feasible technique with acceptable level of safety in the context of vasospastic angina (107, 108) or MINOCA (29, 109). Most recently, Montone et al. (110) reported that Ach provocation test had a low risk of mild and transient complications, with a similar prevalence in both INOCA and MINOCA. Besides, provocation test can help to identify the underlying specific mechanisms of MINOCA and provide individualized pharmacological therapy and prognostic information (109). Hence, it is critical to use provocation test, when coronary spasm is suspected, to identify high-risk patient subgroup.

In fact, different pathophysiologic mechanisms might concurrently exist in the process of MINOCA, therefore, techniques simultaneously provide anatomical and functional information are of great promising. Fortunately, we now have the technique of OFR and UFR, which allow us to get the information of flow reserve, stenosis degree and plaque property at the same time. To the best of our knowledge, no studies have investigated the role of OFR or UFR in MINOCA, it might be a breakthrough to use OFR or UFR to concurrently detect the underline causes and guide the therapy of MINOCA. Furthermore, with the development of technology, the combination of QCT/IVUS with IMR or CFR might also realize in the near future. We believe that more precise therapeutic strategy would be established and applied in MINOCA with the help of more advanced coronary functional assess devices.

## Conclusion and perspective

Numerous studies have demonstrated physiological indices-guided coronary intervention is superior to angiography-based strategy in patients with obstructive CAD (51, 64, 111–113). While in non-obstructive CAD patients, coronary functional assessment provides more precise and individualized treatment as well. Firstly, coronary functional indices, both invasive (e.g., FFR, iFR and QFR) and non-invasive (FFR_CT_), can identify truly non-obstructive CAD among those mild to moderate angiographically stenosis (generally 30–50% according to eyeballing). Secondly, functionally assessment, including wire-based (i.e., CFR, IMR), non-wire-based (Angio-IMR and caIMR) and imaging-based (TTDE, PET and CMR) can evaluate the microcirculatory function, which is regarded as a major mechanism for non-obstructive CAD. Thirdly, with the help of provocation test, vasospastic angina can be identified and treatment targeting coronary spasm will be established. Lastly, anatomically and physiologically combined technologies (OFR and UFR) allow us to simultaneously acquire the information of lumen structure, plaque property, and flow reserve, consequently help us to further understand the underling mechanisms of non-obstructive CAD (especially MINOCA) and to develop specific therapeutic strategies.

Despite the current guidelines recommend the use of FFR/iFR to identify non-obstructive CAD with moderate stenosis, besides, the diagnosis of INOCA is depend on the measurement of CFR, IMR, HMR and vasoreactivity test (4, 14). Several disadvantages, including the use of guidewire, additional expenditure, prolonged time, the need of hyperemic medicine and procedure-related complications, limit the routine use of these functional assessment methods. Hence, non-invasive or relative “non-invasive” methods, for example, FFR_CT_, QFR and Angio-IMR, might provide more promising and attractive utility in clinical practice. Furthermore, in the near future, the microcirculatory resistance perhaps can be assessed by CT, truly realizing the possibility of non-invasive-measured IMR. Nevertheless, it is worth to note that these (potential) techniques are largely dependent on the imaging quality of invasive coronary angiography or computed tomography angiography (CTA). More clinical trials with larger samples and long-term follow-up are needed to investigate the validity of these novel techniques in patients with non-obstructive CAD.

In conclusion, a comprehensive functional assessment is important to identify the underlying mechanism of myocardial ischemia and consequently provide an individualized and appropriate treatment strategy.

## Author contributions

CZ and HF drafted the manuscript. YC revised the manuscript. YZ searched the literature. LS designed the study and edited the manuscript. All authors have read and approved the content of the manuscript.

## Funding

This research was funded by the Key Medicine Disciplines Co-construction Project of Jiaxing Municipal (Grant No. 2019-ss-xxgbx), Jiaxing Science and Technology Program (Grant No. 2021AD30060), Pioneer Innovation Team of Jiaxing Institute of Atherosclerotic Diseases (Grant No. XFCX-DMYH), and Jiaxing Key Laboratory of arteriosclerotic Diseases (Grant No. 2020-dmzdsys).

## Conflict of interest

The authors declare that the research was conducted in the absence of any commercial or financial relationships that could be construed as a potential conflict of interest.

## Publisher's note

All claims expressed in this article are solely those of the authors and do not necessarily represent those of their affiliated organizations, or those of the publisher, the editors and the reviewers. Any product that may be evaluated in this article, or claim that may be made by its manufacturer, is not guaranteed or endorsed by the publisher.
